# Supplementary material for: Co-Precipitated Ni-Mg-Al Hydrotalcite-Derived Catalyst Promoted with Vanadium for CO2 Methanation
Source: Molecules. 2021 Oct 28;26(21):6506. doi: 10.3390/molecules26216506 (PMC8588090; doi:10.3390/molecules26216506)
Supplement: Supplementary file 1 [file molecules-26-06506-s001.zip › molecules-1411078-supplementary.pdf]

molecules

Supplementary information

Co-precipitated Ni-Mg-Al hydrotalcite-derived catalyst promoted with vanadium for CO_2 methanation

Paulina Summa [1, 2, *], Katarzyna Świrk [3], Dominik Wierzbicki [2, 4], Monika Motak [2], Ivo Alxneit [4], Magnus Rønning[3] and Patrick Da Costa [1]

[1] Sorbonne Université, Institut Jean Le Rond d'Alembert, CNRS UMR 7190, 2 place de la gare de ceinture, 78210, Saint-Cyr-L'Ecole, France;
[2] AGH University of Science and Technology, Faculty of Energy and Fuels, Al. A. Mickiewicza 30, 30-059 Kraków, Poland;
[3] Norwegian University of Science and Technology (NTNU), Department of Chemical Engineering, 7491 Trondheim, Norway;
[4] Paul Scherrer Institut (PSI), 5232 Villigen PSI, Switzerland
* Correspondence: patrick.da_costa@sorbonne-universite.fr

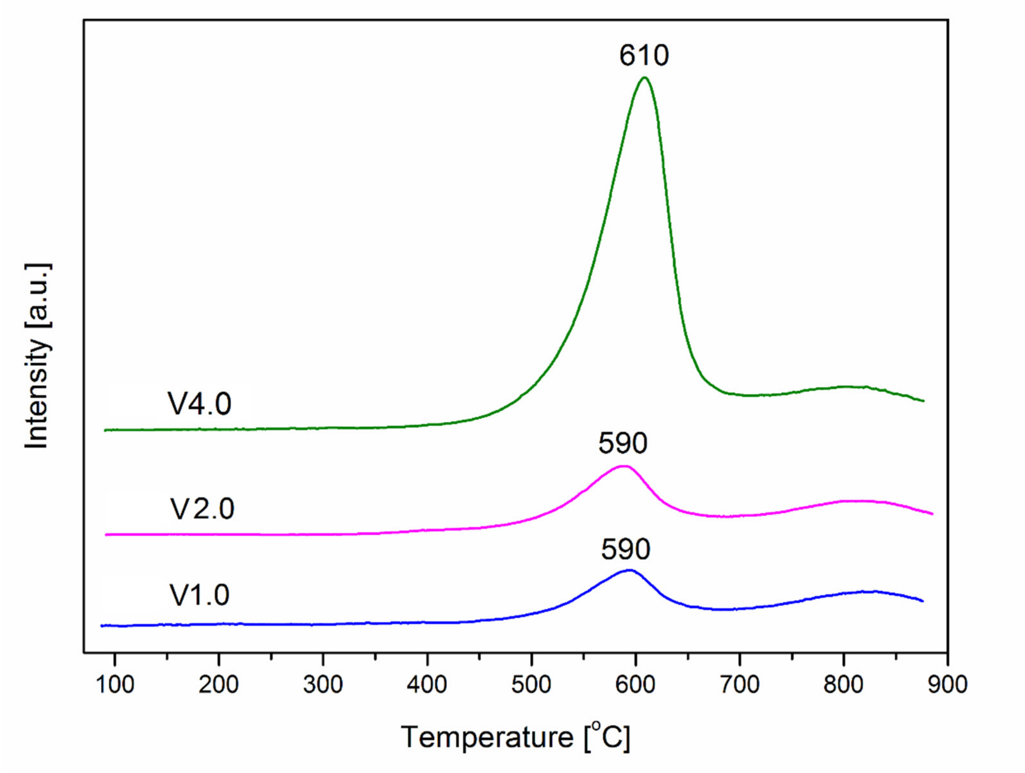

Figure S1. H_2-TPR profiles of the calcined V-Mg-Al hydrotalcite-derived supports.

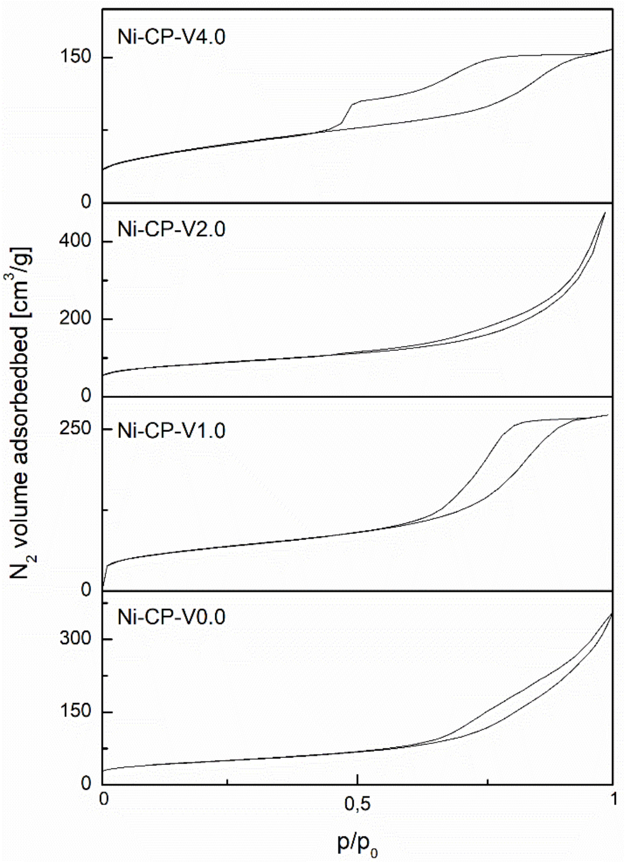

Figure S2. Low-temperature N_2 sorption isotherms for calcined hydrotalcites.

In the following figures elemental maps of the main elements present in the catalysts are reported. These maps were obtained by summing up the EDX signal of several frames acquired in sequence. In each figure at top/right a 2D histogram of the shifts of the individual frames with regard to the first one is reported. The shift was calculated from the 2D cross-correlation of maps of the total X-ray intensities. Generally, shift of only very few pixels are observed indicating that the sample did not move during data acquisition or that the algorithm correcting for sample movement does work well. Bottom/left the time evolution of the integrated X-ray intensity of the carbon Kα line is reported as indicator of the extent of carbon writing during acquisition which could lead to some blurring of small features. The rest of the figures show the spatial distribution of the different elements. Note, that integrated X-ray counts are reported and not concentrations. HAADF images of the identical area are reported in all figures to provide a scale bar and to help with the interpretation of the maps.

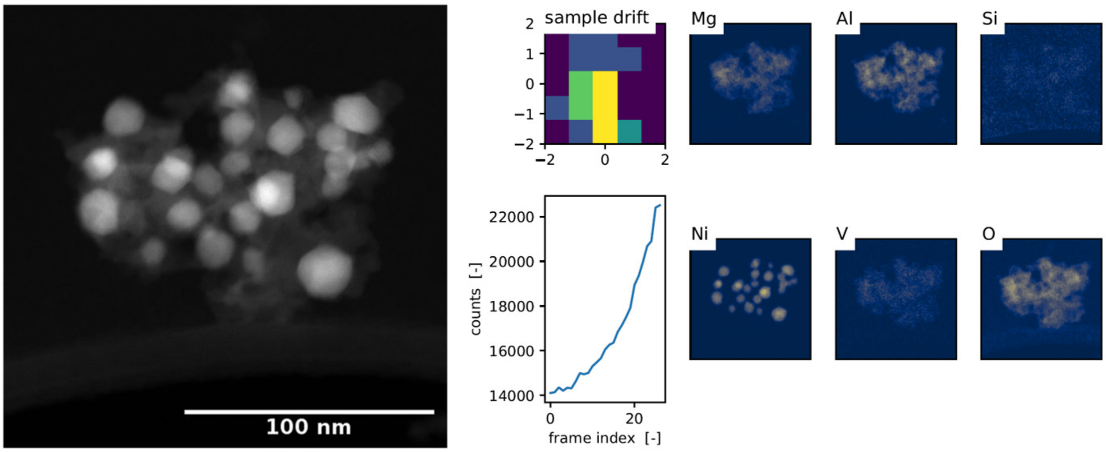

Figure S3. HAADF and EDX for reduced Ni-CP-V2.0.

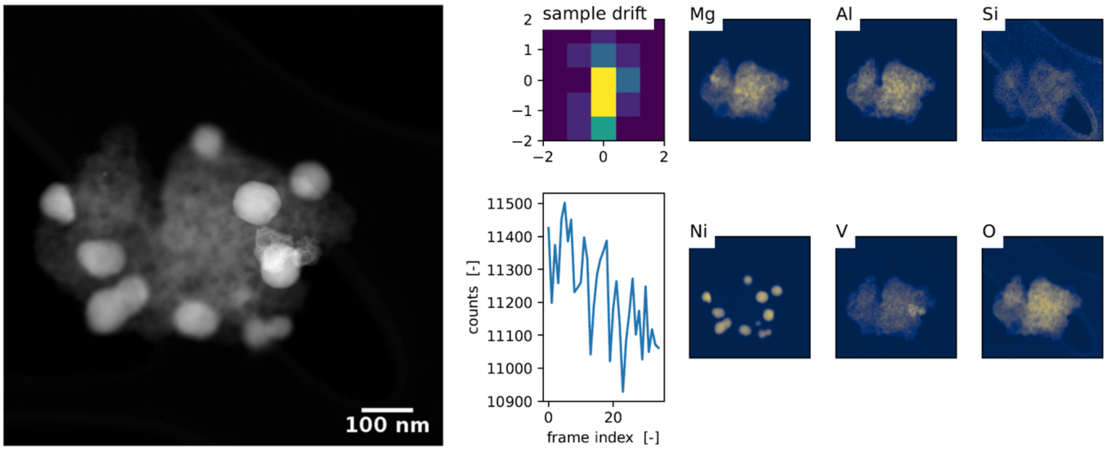

Figure S4. HAADF and EDX for reduced Ni-CP-V4.0.

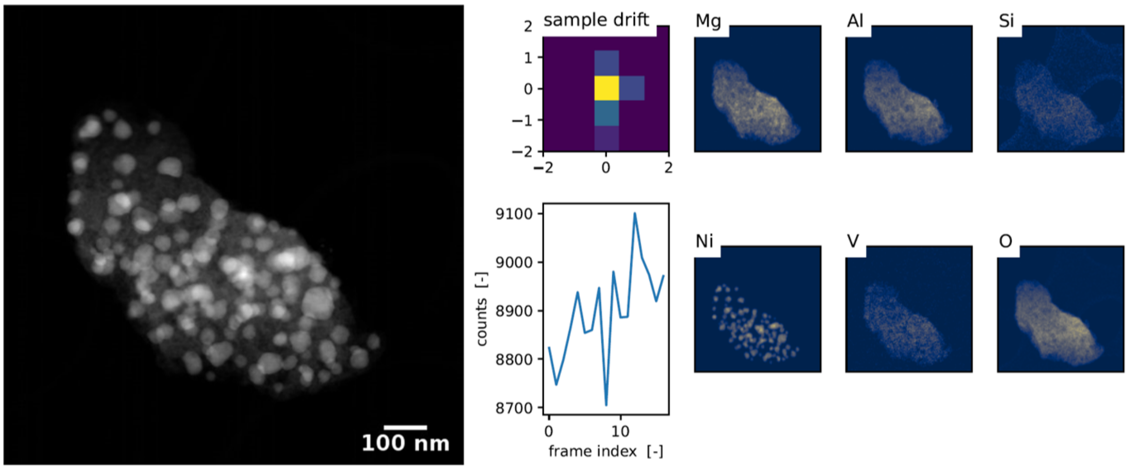

Figure S5. HAADF and EDX for spent Ni-CP-V1.0.

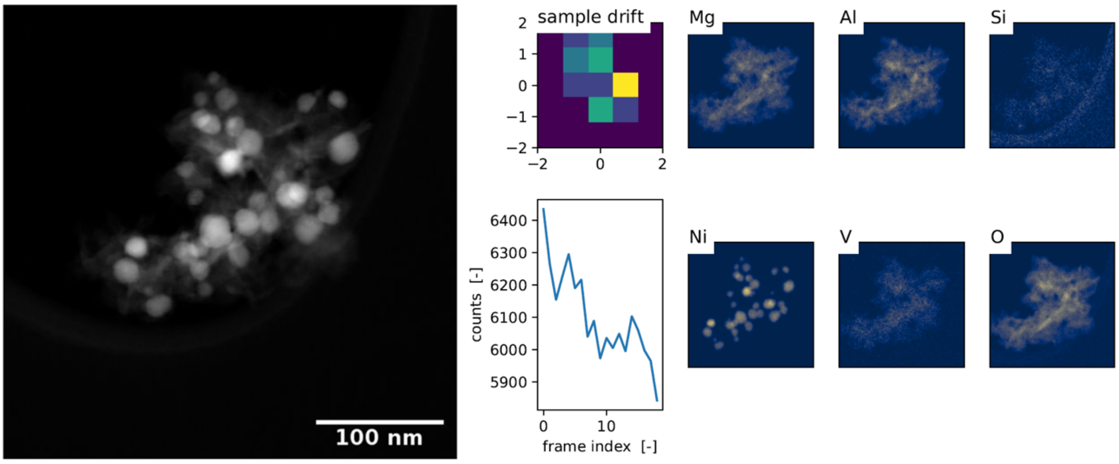

Figure S6. HAADF and EDX for spent Ni-CP-V2.0.

TOF was calculated based on the equation SI Eq. 1

$$\mathrm{TOF} = \frac{\mathrm{r_{CO2}} \cdot \mathrm{M_{Ni}}}{\mathrm{f_{Ni}} \cdot \mathrm{D}} \; [s^{-1}]$$

1

Copyright: © 2021 by the authors. Licensee MDPI, Basel, Switzerland. This article is an open access article distributed under the terms and conditions of the Creative Commons Attribution (CC BY) license (https://creativecommons.org/licenses/by/4.0/).

Where MNi is the molecular weight of nickel [g/mol], f_{Ni} is the metal Ni weight fraction, D is nickel dispersion calculated by HRTEM and rCO_2 is the average reaction rate of CO_2. rCO_2 was calculated via the equation Si Eq. 2.

$$\mathrm{r_{CO2}} = \frac{\mathrm{XCO_2} \cdot \mathrm{F} \cdot (\mathrm{CO_2})}{\mathrm{w} \cdot 22.4 \cdot 10^3 \cdot 60} \cdot \frac{273.15}{298.15} \; [mol \cdot s^{-1} \cdot g_{cat}^{-1}] \quad 2$$

Where F is total used flow rate [ml/min], (CO_2) is the volumetric concentration of carbon dioxide, w is the mass of catalyst [g].

The dispersion was calculated according to the equation SI Eq.3.

$$\mathrm{D} = 6(\frac{\mathrm{v_m}}{\mathrm{a_m}})/\mathrm{d_{va}} \quad 3$$

Where V_m is the volume occupied by an atom in bulk metal (10.95 Å3 for Ni), am is the area occupied by a surface atom (6.51 Å2 for Ni) and d_{va} is mean diameter or metal particle from HRTEM.

References

[1] K. Świrk, J. Grams, M. Motak, P. Da Costa, and T. Grzybek, "Understanding of tri-reforming of methane over Ni/Mg/Al hydrotalcite-derived catalyst for CO2utilization from flue gases from natural gas-fired power plants," *J. CO2 Util.*, 2020.

[2] G. Ertl, H. Knözinger, and J. Weitkamp, "Handbook of Heterogeneous Catalysis," *Handb. Heterog. Catal.*, vol. 1–5, pp. 1–2497, 2008.
